# MINDWALC: mining interpretable, discriminative walks for classification of nodes in a knowledge graph

**DOI:** 10.1186/s12911-020-01134-w

**Published:** 2020-12-14

**Authors:** Gilles Vandewiele, Bram Steenwinckel, Filip De Turck, Femke Ongenae

**Affiliations:** IDLab, Ghent University – imec, Technologiepark-Zwijnaarde 126, Ghent, 9000 Belgium

**Keywords:** Knowledge graphs, Data mining, Explainable AI, Decision tree, Random forest, Feature extraction

## Abstract

**Background:**

Leveraging graphs for machine learning tasks can result in more expressive power as extra information is added to the data by explicitly encoding relations between entities. Knowledge graphs are multi-relational, directed graph representations of domain knowledge. Recently, deep learning-based techniques have been gaining a lot of popularity. They can directly process these type of graphs or learn a low-dimensional numerical representation. While it has been shown empirically that these techniques achieve excellent predictive performances, they lack interpretability. This is of vital importance in applications situated in critical domains, such as health care.

**Methods:**

We present a technique that mines interpretable walks from knowledge graphs that are very informative for a certain classification problem. The walks themselves are of a specific format to allow for the creation of data structures that result in very efficient mining. We combine this mining algorithm with three different approaches in order to classify nodes within a graph. Each of these approaches excels on different dimensions such as explainability, predictive performance and computational runtime.

**Results:**

We compare our techniques to well-known state-of-the-art black-box alternatives on four benchmark knowledge graph data sets. Results show that our three presented approaches in combination with the proposed mining algorithm are at least competitive to the black-box alternatives, even often outperforming them, while being interpretable.

**Conclusions:**

The mining of walks is an interesting alternative for node classification in knowledge graphs. Opposed to the current state-of-the-art that uses deep learning techniques, it results in inherently interpretable or transparent models without a sacrifice in terms of predictive performance.

## Background

### Introduction

Graphs are data structures that are useful to represent ubiquitous phenomena, such as social networks, chemical molecules, biological protein reactions and recommendation systems. One of their strengths lies in the fact that they explicitly model interactions between individual units (i.e. nodes) in the form of edges [1], which enriches the data. Today, graphs are increasingly being leveraged for various machine learning tasks [2]. For example, one might recommend new friends to a user in a social network [3], predict the role of a person in a collaboration network [4], or classify the role of a protein in a biological interaction graph [5]. Knowledge graphs (KG) are representations of domain or expert knowledge encoded as a collection of triples having the form (subject, predicate, object). These triples can be directly mapped onto a named edge (the predicate) linking together two named nodes (the subject and object). KGs have been gaining a lot of attention, as many of them, such as YAGO [6], DBpedia [7], NELL [8], Freebase [9], and the Google Knowledge Graph [10], have already been successfully applied to various real-world applications.

Recently, the use of deep learning techniques to either learn representations of nodes in the graph, or to directly learn a model for the task at hand, has been gaining immense popularity. While these techniques achieve good predictive performances, they can be considered black-box and thus lack interpretability. The explainable and transparent aspects of a predictive model are of vital importance for applications situated in critical domains, such as health care and finance, as a wrong decision could have significant negative repercussions. Therefore, a new shift of focus within research towards explainable AI is taking place [11, 12]. Currently, techniques exist that can give post-hoc local explanations for a black-box model’s predictions of certain samples, such as which features contributed most towards giving a certain prediction [13, 14]. Unfortunately, these techniques are not able to deliver a global explanation, making it infeasible to grasp all the internals of the black-box model. Moreover, they exhibit other weaknesses such as susceptibility to adversarial attacks [15]. In contrast to making black-box techniques more transparent, we could instead focus on using inherently interpretable techniques, especially for critical domain applications [16].

Classical machine learning approaches, such as Random Forest (RF) and Logistic Regression (LR), require a numerical representation of the data (in the form of a matrix) as input. As a graph itself is not numerical, an intermediary step is required that transforms the nodes in our graph into numerical vectors. In this paper, we introduce an algorithm that generates a numerical representation for the nodes in a graph. It does this by efficiently mining for graph substructures of a specific type. These substructures are informative for an entity belonging to a specific class, when found in its neighborhood. Moreover, we present three different approaches to combine with our mining algorithm to classify nodes or entities in a KG. First, we apply the algorithm recursively in order to induce a decision tree. The resulting model is fully interpretable, since we can easily visualize the entire tree or highlight the taken path in our decision tree to form a prediction. We demonstrate this by inspecting and discussing induced decision trees on benchmark data sets. Second, we induce multiple decision trees, each with a subsample of both training entities and possible substructures, in order to form a forest. This ensemble technique often results in better predictive performances, but comes at a cost of lower interpretability and longer training time. Finally, we decouple the modeling and mining by performing a single pass over the data to mine a collection of informative walks. These walks can then be used to create high-dimensional binary feature vectors that can be passed to any classification algorithm. This final technique is fast, as it requires only a single pass over the data. It also achieves high predictive performances, as we will show empirically. Nevertheless, even when used in combination with interpretable techniques, such as LR, the interpretability can be considered the lowest of all three techniques due to its high dimensionality.

The remainder of the paper is organized as follows. In the next section, we discuss some related approaches that are used to tackle the problem of node classification in KGs, and what their shortcomings are. In “Context”, we provide the necessary background to discuss, in “Methodology”, the different steps taken to mine informative graph substructures and how they can be used for classification. Then, in “Results”, we elaborate on the setup we used for different experiments and provide the obtained results. We discuss these results in “Discussion”. Finally, we conclude our paper and provide future research directions in “Conclusion and future work” sections.

### Related work

Different types of approaches can be identified in order to create predictive models using KGs. A first type of approaches are classical ones. Here, information about the structure of the graph is explicitly encoded into a feature vector, which can then be fed to a machine learning model [17]. Examples of such features are indications of the presence of local neighborhood structures [18] and graph statistics [19]. When features that make sense to humans are used within the pipeline, these approaches can be classified as being interpretable if the features are fed to a white-box model. Unfortunately, the disadvantage of this type of approach is that it is not task-agnostic: they need to be tailored specifically for the task and application domain at hand. This results in an increased creation effort. Another popular classical approach, which is more task-agnostic, is applying kernel methods [20]. These methods measure the similarity between two knowledge bases, either directly on their graph representation [21–23] or based on description logics [24]. Unfortunately, using pairwise similarity measures as features is often less interpretable than using human-understandable variables.

A second type of approach, which has been gaining immensely in popularity, is representation learning, often known as embedding techniques. The goal of representation learning is to map the graph-based structures onto a low-dimensional numerical vector that can be used for downstream machine learning tasks [25]. One possibility to create these numerical vectors is by applying matrix or tensor factorization. These methods represent the KG in a large 3-dimensional binary matrix, which is then factorized into different vectors [26]. Another possibility is to build on popular unsupervised deep learning techniques, such as Word2Vec [27]. Here, the sentences that are normally fed to Word2Vec are replaced by walks taken in the graph. These walks can either be completely random [28], or guided by some metric, called biased walks [29, 30]. Representation learning can be seen as completely task-agnostic since representations can be reused for multiple tasks. Also, these techniques often tend to achieve higher performances than, for example, their kernel or classical feature-based counterparts. The disadvantage of these approaches is that by mapping an entity to a low-dimensional latent representation, all interpretability is lost.

A final and very recent type of approach involves adaptations of neural networks that can directly work on graph-based data [31, 32], which have already been successfully applied to KGs [33]. Again, these techniques can be seen as black-boxes, making it very hard or even impossible to extract any insights from the model. In this work, the objective is to design a technique that resembles the predictive power of black-box approaches, while allowing for explainability.

### Context

In this section, we first explain some fundamental concepts and notation, which will be used in the remainder of this work.

#### Entity classification: problem definition

Given a multi-relational directed KG $\mathbb {G} = (\mathbb {V}, \mathbb {E}, \ell)$, constructed from a collection of triples, where $\mathbb {V}$ are the vertices or entities in our graph, $\mathbb {E}$ the edges or predicates and *ℓ* a labeling function that maps each vertex or edge on its corresponding label. Moreover, we are provided with a data set ***D***=(***V***, ***y***), with ***V*** a list of vertices and ***y*** their corresponding labels. We shall denote a specific training vertex or entity as *v*_*i*_ and its corresponding label with *y*_*i*_. Our goal is to construct a model or hypothesis *h*(.) based on **V** that minimizes a loss function $\mathcal {L}$(.) to **y**, and which generalizes well to unseen vertices:

1 $$ \underset{h}{{argmin}}\ \, \mathcal{L}(\boldsymbol{y}, h(\boldsymbol{V}))  $$

#### Converting KGs

**Fig. 1:**
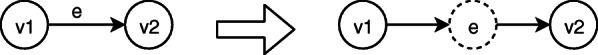
Converting a triple consisting of two labeled nodes and a labeled edge to three labeled nodes


As done by de Vries et al. [23], we first simplify the KG by removing its multi-relational aspect. To do this, we represent each (subject, predicate, object) triple from the original KG as three labeled nodes and two unlabeled edges (subject → predicate and predicate → object), as depicted in Fig. 1. This transformation reduces the complexity of the further elaborated procedures, without a loss of correctness, since a distinction between entities and predicates is no longer needed.

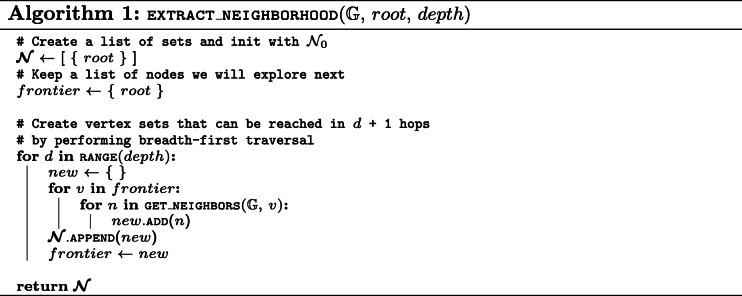


#### Neighborhoods, walks and wildcards

We characterize each instance *v*∈***V***, by its neighborhood $\boldsymbol {\mathcal {N}}(v)$ of a certain depth *d*. The neighborhood is a subgraph that contains all vertices that can be reached by traversing *d* edges from the instance *v*. It can be extracted, for example, by performing a breadth-first-traversal.

We define a walk as a sequence of vertices. The first vertex within this walk is often called the root of the walk. This root can be seen as a placeholder, which is replaced by a specific vertex depending on the context. We denote a walk as root→*v*_*0*_→*v*_*1*_→*v*_*2*_→….

We introduce a new special type of hop for our walks, which we call a ‘wildcard’ and notate by an asterisk ∗. The interpretation of this wildcard is that any edge or vertex label can be matched on that position in the sequence. This enables the walks to have more expressive power. To illustrate this, imagine that the presence of an entity of a specific type $\mathcal {T}$ is very discriminative for a certain class. It is possible that only the fact that this entity is of that type carries information, while the specifics of the entity itself are unimportant. As such, this could be represented by a walk root→∗→∗→rdf:$\mathtt {type \rightarrow \mathcal {T}}$.

## Methodology

In the following section, we elaborate upon the steps of our walk mining algorithm and show three different techniques to use in combination with the mining algorithm to classify entities.

### Discriminative walk mining

In this study, we will focus on a special type of walk. A walk of depth *l* has a root placeholder, followed by *l*−1 wildcards and ending in a specific vertex with label *x*:

2 $$ \mathtt{root \rightarrow} \underbrace{\mathtt{* \rightarrow... \rightarrow *}}_{l-1} \mathtt{\rightarrow x}  $$

As mentioned, the first hop, root, is replaced by *v* whenever we want to search for it in its neighborhood $\boldsymbol {\mathcal {N}}(v)$. Alternatively, we can represent these walks by a tuple: *w*=(*v*, *l*), which is the notation we will use for the remainder of this work. When extracting a neighborhood of depth *d*, we keep track of *d* different sets $\left \{\boldsymbol {\mathcal {N}}_{i}(v)\ |\ 1 \leq i \leq d \right \}$, where $\boldsymbol {\mathcal {N}}_{i}(v)$ stores the nodes that can be reached in exactly *i* hops. Whenever we want to search for a certain walk *w*=(*v*, *l*) in a neighborhood, we only need to check whether *v* appears in $\boldsymbol {\mathcal {N}}_{l}(v)$. This avoids the need to traverse parts of the graph. Due to the nature of our walk and the use of this data structure, we are able to search for these types of walks in a neighborhood in constant time. Moreover, these types of walks already possess a rich amount of expressive power, as we will demonstrate further empirically.

Our goal is to mine a walk *w*=(*v*, *l*) that maximizes information gain (IG) on a given data set ***D***:

3 $$ \underset{w}{\text{argmax}}\ \, IG(\boldsymbol{D},\ w)  $$

For each candidate walk, we can calculate its mutual information or information gain [34], on a given data set ***D***. This is defined as the weighted reduction in entropy obtained by partitioning the data:

4 $$ IG(\boldsymbol{D},\ w) = H(\boldsymbol{D}) - H(\boldsymbol{D}\ |\ w)  $$

where *H*(***D***) is called the prior entropy, and *H*(***D*** | *w*) the conditional entropy of the data obtained by partitioning the data based on (the presence of) *w*. We can calculate the (prior) entropy of a data set ***D***=(***V***, ***y***) using its label vector consisting of discrete labels *y*_*i*_∈{1,…,*C*}, with *C* the number of classes:

5 $$ H(\boldsymbol{D}) = -\sum\limits_{k=1}^{C} p_{k} * \log{p_{k}}  $$

with *p*_*k*_ the fraction of labels having value *k* in ***y***:

6 $$ p_{k} = \frac{1}{|\boldsymbol{y}|} \sum\limits_{i=0}^{|\boldsymbol{y}| - 1} \mathbbm{1} (y_{i} = k)  $$

*𝟙* being the identity function which is equal to 1 in case the condition is true, else it is 0. To calculate the entropy conditioned on a walk, *H*(***D*** | *w*), we first partition our data. One partition consists of labels corresponding to vertices for which the walk can be found in its neighborhood. The other partition consists of labels corresponding to vertices for which the walk cannot be found:

7 $$ \begin{aligned} \boldsymbol{D_{f}} = \{ (v_{i},\ y_{i})\ |\ w \in \boldsymbol{\mathcal{N}}(v_{i}) \} \\ \boldsymbol{D_{nf}} = \{ (v_{i},\ y_{i})\ |\ w \notin \boldsymbol{\mathcal{N}}(v_{i}) \} \end{aligned}  $$

After partitioning, we can calculate *H*(***D*** | *w*) as follows:

8 $$ H(\boldsymbol{D}\ |\ w) = \frac{|\boldsymbol{D_{f}}|}{|\boldsymbol{D}|} * H\left(\boldsymbol{D_{f}}\right) + \frac{|\boldsymbol{D_{nf}}|}{|\boldsymbol{D}|} * H\left(\boldsymbol{D_{nf}}\right)  $$

### Example

**Fig. 2:**
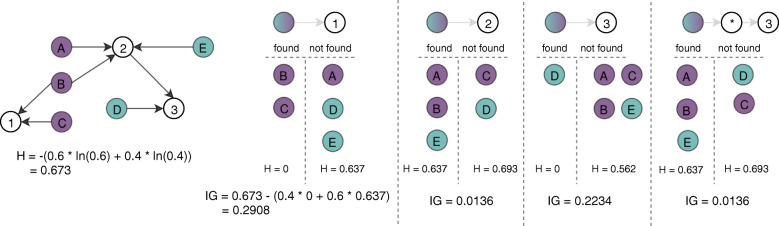
An example of our walk mining algorithm on a simple artificial graph

To further clarify our algorithm, we provide an example of a binary classification problem using a simple artificial graph in Fig. 2. The nodes in the graph with a letter have a corresponding label or class (its color), while the nodes with a number are unlabeled vertices. The walk maximizing the information gain in this example is root -> 1 or (1, 1).

### Implementation

We now present pseudo-code for the mining algorithm. It consists of three different procedures: (i) EXTRACT_NEIGHBORHOOD (Algorithm 1) will create the data structure for each training vertex in order to test for the presence of a certain walk efficiently, (ii) INFO_GAIN (Algorithm 2) will calculate the information gain of a walk for the provided training neighborhoods and labels, and (iii) MINE_WALKS (Algorithm 3) is the main procedure that uses the two other procedures to mine the *n* most informative walks.


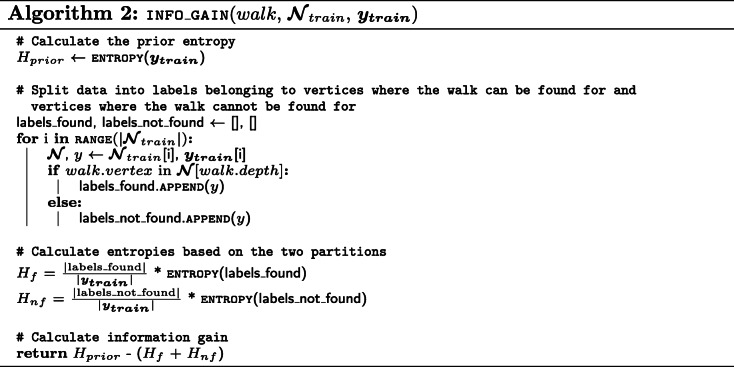


It is important to note that, due to the conversion discussed in “Converting KGs” section, (*v*, *l*) candidates, with *l* being an odd value, correspond to predicates in the original KG. Therefore, to mine walks that require *k* hops in the original KG (i.e. visit *k* entities), the *depth* parameter needs to be set to *k*∗2. Additionally, candidates with odd depths can often be skipped in Algorithm 3 as the presence or absence of predicates often carries little to no information.

### Computational complexity

The algorithm itself will calculate the information gain for each possible (*v*, *l*) combination. In total, there are |***V***| vertices, and the maximal depth of the walks, *d*, is a hyper-parameter of the algorithm. As such, there are $\mathcal {O}(d|\boldsymbol {V}|)$ possible walk candidates. In order to calculate the information gain of a candidate, we have to test for its presence in all the training neighborhoods. This scales linearly in function of the number of training instances |***V***_*train*_|. As such, the total computational complexity to mine the most informative walk, and in addition calculate the information gain of all other candidates, is equal to $\mathcal {O}(d|\boldsymbol {V}_{train}||\boldsymbol {V}|)$. It should be noted that the number of training entities, |***V***_*train*_|, is often much smaller than the number of entities in the entire graph, |***V***|. As such, the complexity scales linearly in function of the total number of vertices in the graph.


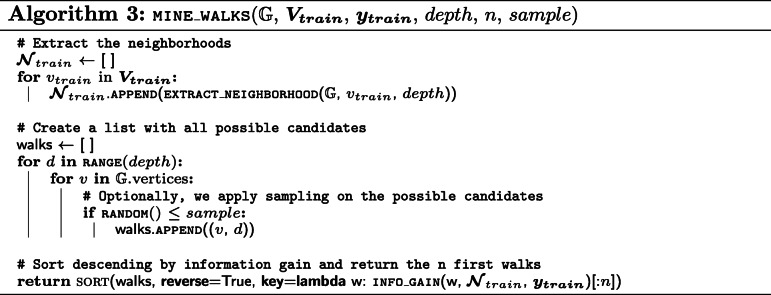



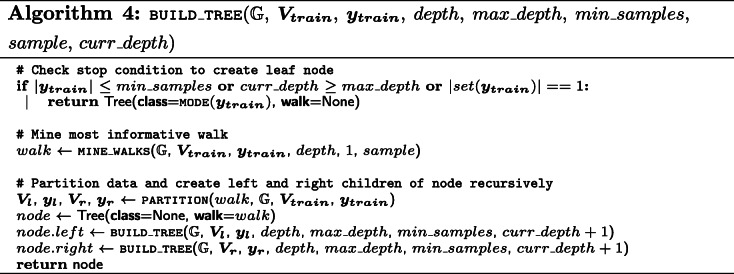



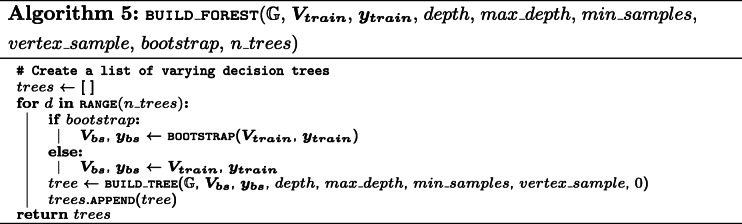


### Classification with discriminative paths

We described how to mine a walk that maximizes information gain. Often, one walk is not enough to create a perfect separation between the different classes in the feature space, especially when dealing with a multi-class problem. Therefore, we propose three different techniques to combine with our proposed walk mining algorithm. Each of the presented techniques has different characteristics, making them ideally suited for different use cases.

#### Decision tree induction

One straightforward approach is to mine these walks recursively in order to create a decision tree. In each iteration, we mine the most discriminative walk. After this, we partition our data into a collection of instances for which the walk can be found in its neighborhood, and a collection of instances for which this walk cannot be found. These two partitions form the left and right child of a node respectively. We then continue recursively on these two new child nodes, until the labels of a certain partition are all from the same class (stop condition) at which point we create a leaf node for the tree. Examples of such decision trees are provided in “Interpretable characteristics” section.

While decision trees possess excellent interpretability characteristics, they can be prone to over-fitting [35]. Therefore, two hyper-parameters are introduced that allow for pre-pruning, which halts the algorithm preemptively by extending the stop condition. On the one hand, the algorithm halts when a certain depth (max_depth) is reached. On the other hand, the algorithm stops when the number of samples in a particular node of the decision tree is lower than a specified number (min_samples). The pseudo-code for this technique is depicted in Algorithm 4 We call the procedure for building a single tree that tests all possible walk candidates, by setting *sample* equal to 1.0 and *curr*_*depth* to 0.

It should be noted that our proposed induction approach shares a lot of similarities with already existing algorithms such as CART [36] and C5.0 [37]. These algorithms work on feature matrices and recursively mine for the most informative feature to induce a tree. Finding the most informative feature is done by calculating a splitting criterion such as information gain or Gini coefficient for all possible feature and threshold combinations. Our technique replaces this phase where the most informative feature is sought, by mining the most informative walk. This allows our algorithm to work directly on graph data.

#### Extending to RF

Decision trees are often able to achieve high predictive performances, while being completely interpretable. However, they can be susceptible to a high variance or over-fitting. A RF is a technique that reduces the variance by creating an ensemble of decision trees, in which each tree is constructed from a fraction of training instances and features. This often results in an increase in predictive performance, as has been shown empirically [38]. In our implementation, as shown in Algorithm 5, the type and amount of sampling can be controlled through two hyper-parameters. To construct each tree using different weightings of the samples, the bootstrap parameter can be set to true, which will sample |***D***| times with replacements from ***D***. To make sure each tree uses different features, the vertex_sample parameter can be used, which is a value between 0 and 1 and which corresponds to the fraction of candidates that are randomly sampled to create each internal node of the decision trees.

While an ensemble of decision trees often results in a better predictive performance, this comes at the cost of lower interpretability and higher computational runtime for both training and inference. The loss of interpretability is due to the fact that different trees need to be studied in order to grasp the model. Nevertheless, some interpretability is still present, as the most important features for the model can easily be listed. This can be done by counting how many times a certain walk is used in the different decision trees of the ensemble, additionally taking into account the position of the walk in the tree (as a root node is often more important than a node at a higher depth), which we will show in “Interpretable characteristics” section. The computational runtime scales linearly in function of the number of trees in the ensemble. The pseudo-code for this approach is presented in Algorithm 5.


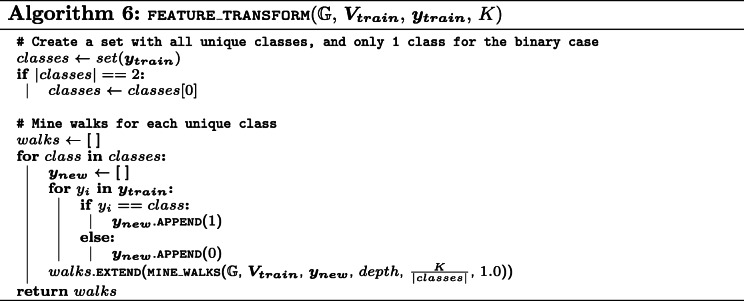


#### Feature transform

**Fig. 3:**
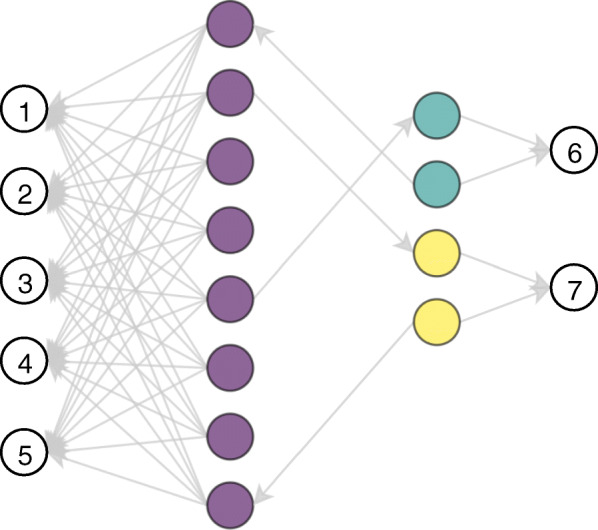
A simple artificial example where 6 walks would have to be extracted by the feature transform approach in order to obtain perfect separation, while the tree-based approaches only need 2

By performing a single pass over all possible walk candidates and keeping track of the *K* highest-scoring walks, we can decouple the walk mining from the model induction. This is done by using the *K* mined walks to create *K*-dimensional binary feature vectors for the training and testing instances and feeding these to any modeling technique. Each feature in this vector corresponds to the presence of a certain walk in an instance’s neighborhood. The advantage of this approach is that the runtime is low, since only a single pass over the data has to be performed. The disadvantage is that the information gain for each of these candidates will be calculated on the entire data set, as opposed to specific partitions of the data set, as happens for the tree-based techniques. Especially for imbalanced data sets, only performing a single pass could result in favoring the walks that are only able to distinguish between the majority class and all other classes. To illustrate this, we created a very simple graph that is depicted in Fig. 3. The network represents a three-class classification problem that is imbalanced, as the purple class has eight samples while the yellow and green class only have two samples. Clearly, two walks would be enough to have perfect separation between all three classes: {(*v*_1_,1),(*v*_2_,1)} with *v*_1_∈{1,2,3,4,5} and *v*_2_∈{6,7}. The decision tree approach would first mine one (*v*_1_,1) as the information gain is highest for those walks, and then partition the data into a data set with only purple nodes and a data set with the two yellow and green nodes. Afterwards, it would mine the (*v*_2_,1) in the latter partition as the information gain would be highest in that partition of the data. As such, due to the partitioning, walks are mined that are discriminative for specific parts of the data. In contrast, we would have to set *K*>5 to obtain perfect separation, as the information gain of all {(*v*_1_,1) | *v*_1_∈{1,2,3,4,5}} is higher than {(*v*_2_,1) | *v*_2_∈{6,7}} in the context of the entire data set.

To combat this, we perform *C* passes over multi-class data instead, by mapping the targets ***y*** onto binary vectors ***y***_***k***_ with *k*∈{1,…,*C*} to mine $\frac {K}{C}$ walks:

9 $$ y_{k,i} = \left\{\begin{array}{cl} 1 & \text{if \(y_{i}=k\)} \\ 0 & \text{else} \\ \end{array}\right.  $$

The pseudo-code for the feature transform approach is listed in Algorithm 6.

## Results

In this section, we will evaluate the three proposed techniques in terms of predictive performance, runtime and interpretability.

### Data sets

We extracted four benchmark data sets, describing KGs, available from a public repository set up by Ristoski et al. [39]. The *AIFB* data set describes scholarly data of 178 researchers in the Institute of Applied Informatics and Formal Description Methods. The goal is to classify for each of these researchers to which of the four research groups they belong. The *BGS* data set, stemming from the British Geological Survey, describes geological measurements of 146 rock units. The goal is to classify whether certain rocks are fluvial or glacial. The *MUTAG* data set describes 340 complex chemical molecules. Here, we need to determine for each of these molecules whether or not they are potentially carcinogenic. Finally, the *AM* data set describes 1000 historical artifacts from the Amsterdam Museum, which need to be classified into one of eleven categories. For each of these data sets, we remove triples with specific predicates that are too correlated with the target from our KG, as provided by the original authors. Moreover, a predefined split into train and test set, with the corresponding ground truth, is provided by the authors, which we used in our experiments. The train set is used to mine the walks and induce the models, which are then evaluated on the test set. We summarize the properties of these data sets in Table 1.

**Table 1:** The properties of the four KG benchmark data sets used within this study

	*AIFB*	*BGS*	*MUTAG*	*AM*
Triples	29,226	916,421	74,567	5,700,371
Entities	8,285	333,863	23,644	1,498,566
Relations	47	105	24	100
Classes	4	2	2	11
Train entities	141	117	272	802
Test entities	37	29	68	198
Label predicates	affiliation	hasLithogenesis	isMutagenic	objectCategory
	employs	hasLithogenesisDescription		material
	carriedOutBy	hasTheme		

### Predictive performance

To assess the predictive performance of our proposed approaches, we compare our three approaches to two well-known techniques: (i) an adaptation of Graph Convolutional Networks (GCN) specifically made for relational data (R-GCN) [40], and (ii) RDF2VEC which learns a representation for the nodes in the graph in an unsupervised, task-agnostic manner [28]. We used the following configurations for each of our approaches:

- For the Tree approach, we tune the maximal depth of the tree using cross-validation on the training set. The possible values for the maximal depth were {3,5,10,*None*}. *None* corresponds to growing trees until there is perfect classification on the training set.
- For the Forest approach, three different hyper-parameters were tuned using cross-validation on the training set: (i) we tuned the maximal depth of the trees in the forest to be either 5 or None, (ii) the amount of vertex sampling to be equal to 0.5 or 0.9, and (iii) the number of trees in the forest to be one of {10,25,50}.
- The Transform approach extracted 10000 walks using the training set in order to transform both training and test set into binary feature matrices. Walks that could only be found for one of the training instances or all training instances were immediately removed. The resulting matrices were then fed to a LR (with *l*_1_ regularization) and RF Classifier. The (inverse of the) regularization strength (*C*) of the LR classifier was tuned to be in {0.01,0.1,1.0,10.0,100.0,1000.0,10000.0}. For the RF classifier the maximum depth of the trees and the number of trees were tuned to be in {5,10,None} and {10,100,250} respectively.

For each data set, we performed 10 runs. The average accuracy scores achieved on the test set and their corresponding standard deviations are summarized in Table 2. The results for the Relational Graph Convolutional Network (R-GCN) and RDF2VEC are taken directly from Schlichtkrull et al. [40].

**Table 2:** The accuracy scores of Relational Graph Convolutional Networks (R-GCN), RDF2VEC and our proposed approaches on four benchmark data sets

data set	R-GCN	RDF2VEC	Tree	Forest	Transf+LR	Transf+RF
AIFB	**95.83 ±0.62**	88.88 ± 0.00	85.83 ± 2.43	90.83 ± 2.29	85.28 ± 1.34	86.39 ± 0.88
BGS	83.10 ± 0.80	87.24 ± 0.89	87.58 ± 1.78	91.38 ± 2.44	89.31 ± 1.09	**93.10 ± 0**
MUTAG	73.23 ± 0.48	67.20 ± 1.24	68.97 ± 4.46	74.41 ± 2.88	**78.82 ± 1.86**	73.24 ± 1.16
AM	89.29 ± 0.35	88.33 ± 0.61	86.81 ± 1.27	88.33 ± 0.73	**90.05 ± 0.05**	89.89 ± 1.14

### Runtime

For each of the accuracy measurements taken in “Predictive performance” section, we also measured the time it took to fit the model. The average fitting times (in seconds) and their corresponding standard deviations for the 10 taken measurements are listed in Table 3.

**Table 3:** The computational runtime (in seconds) required to fit the predictive models for our three proposed techniques on the four KG benchmark data sets

data set	Tree	Forest	Transform
AIFB	24.83 ± 1.04	132.36 ± 63.31	24.22 ± 0.61
BGS	12.41 ± 0.43	65.71 ± 34.49	6.63 ± 0.29
MUTAG	14.51 ± 0.60	104.88 ± 32.84	4.66 ± 0.37
AM	643.29 ± 12.25	7620.88 ± 2419.94	880.96 ± 22.04

### Interpretable characteristics

In this section, we inspect interesting parts of induced decision trees on the different data sets.

#### AIFB

**Fig. 4:**
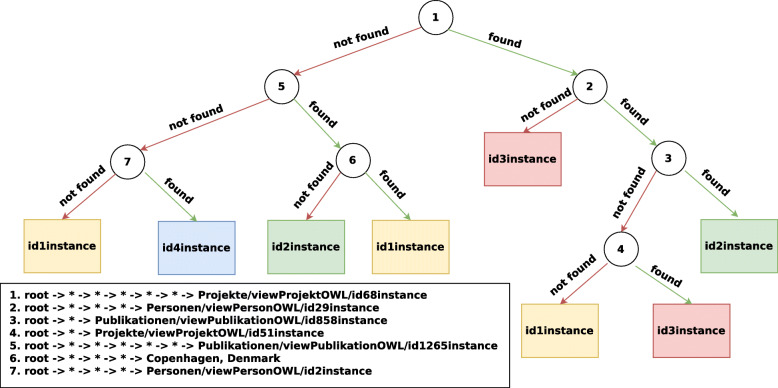
An induced decision tree with a maximum depth of 5, on the *AIFB* data set

For the *AIFB* we set the maximum depth of this decision tree to 5 and the maximum path depth to 6 such that the tree and extracted paths do not become too complex. The accuracy score of the decision tree, presented in Fig. 4, on the predefined test set, is equal to 86.11*%*. In the root node, we find the walk root -> * -> * -> * -> * -> * -> viewProjektOWL/id68instance. When this walk can be found in the neighborhood of an instance, it can no longer be of the research affiliation id4instance, as this leaf does not occur in the subtree on the right. Moreover, this type of walk already demonstrates the added value of having a fixed depth, by the use of wildcards, in our walk. As a matter of fact, we could end up in an entity which is of a type Project in only two hops (e.g. root -> * -> viewProjektOWL/id68instance) from an instance in *AIFB*, but this results in a lot less information gain than when six hops need to be taken. When inspecting the original KG, it appears that only two people, who are both from affiliation id3instance, are directly involved in the Projectid68instance, or in other words where this path with only two hops could be matched. On the other hand, it appears that these two people have written quite a large amount of papers with the other researchers in their affiliation. As such, a walk that first hops from a certain person (the root) to one of his or her papers, and going from there to one of the two people mentioned earlier through an author predicate can be found for 45 people from affiliation id3instance, 3 people from id2instance and 2 people from id1instance. The remaining nodes in the right subtree from the root are less informative, since these will try to split the 5 people from both affiliation id2instance and id1instance from the 45 others.

#### BGS

**Fig. 5:**
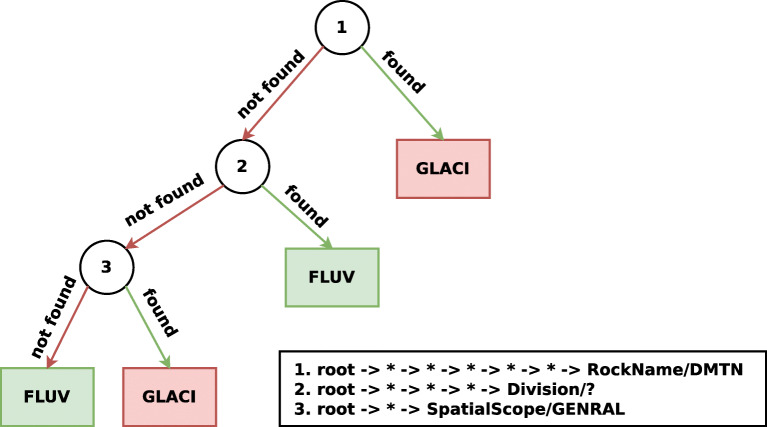
An induced decision tree with a maximum depth of 3, on the *BGS* data set

For the *BGS* we set the maximum depth of this decision tree to 3 and the maximum path depth to 8. The simple tree presented in Fig. 5 achieves an impressive accuracy of 89.66*%*. In the root node, the walk root -> * -> * -> * -> * -> * -> RockName/DMTN can be found. DMTN stands for diamicton [41], which is a term often applied to unsorted glacial deposits, which is informative for the GLACI (glacial) class, as it can be found for 32 out of the 43 glacial training instances. When retrieving walks of depth 6 that end in RockName/DMTN, we find a pattern of an instance hopping through the skos:broader predicate to one out of eleven different geographical groupings present in the KG (e.g. the British Coastal Deposits Group) that have diamicton sediment. When the walk in the root node is not found, the presence for the following walk, is tested: root -> * -> * -> * -> Division/?. The walk is very informative for the FLUV (fluvial) class, which is sediment produced by rivers, with 38 out of 74 fluvial training instances for which it can be found. It reaches Division/? again through a geographical grouping and ends up in the node through the hasPredominantAge predicate. This means that the predominant geological age of the geographical grouping is undefined. The final walk corresponds to root -> * -> SpatialScope/GENRAL, which is rather informative for the fluvial class if the walk is not found, with 27 out of the remaining 36 fluvial samples ending up in this partition, but in combination with 5 out of the 11 remaining glacial samples. SpatialScope/GENRAL corresponds to when the applicability of the KG’s definition is generally applicable.

#### AM

**Fig. 6:**
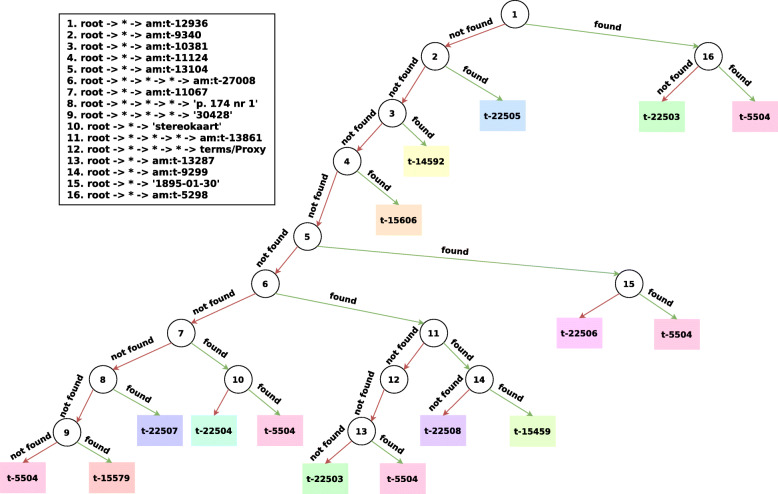
An induced decision tree with a maximum depth of 9, on the *AM* data set. am: is a prefix for the namespace http://purl.org/collections/nl/am/

For the *AM* data set, a higher max depth is required in order to achieve a good predictive performance. This is due to the fact that there are 11 different classes in this data set. We therefore set the maximum depth to 9 and the maximum depths of the paths to 8. The induced decision tree is depicted in Fig. 6. The accuracy on the test set of this tree is 79.29%. When inspecting the depth of the decision trees induced for the results in “Predictive performance” section, we see that a max depth of around 30 is required to achieve good accuracies. Such deep trees are of course somewhat harder to visualize and would require an interactive interface where parts of the tree can be collapsed. It should also be noted that it is always possible to highlight the path taken in the decision tree to generate a prediction, which serves as an excellent local explanation.

If we look at the mined walks, we see that they are very shallow, i.e. there 5 walks of depth 4 and 11 walks of depth 2. As such, the most discriminative information is located in the close neighborhood of the training entities. In the root, we find the walk root -> * -> am:t-12936. If this walk can be found in the neighborhood of a training entity, this is informative for class t-22503. am:t-12936 appears to be associated with the objectName predicate, which can be found for 228 of the 278 training entities of class t-22503 and for 1 of the 102 training entities of t-5504. The walk straight to the right of the node therefore solely exists to isolate the only entity of t-5504 from the others. Then for the first three children on the left-most path of the tree, we find three walks (walk 2, 3 and 4) that are informative for classes t-22505, t-14592, t-15606 in respective order. Walk 5 can be found for all of the training instances of class t-22506 and for 2 of the remaining 101 training entities of class t-5504. Walk 6 then partitions the remaining classes in two large groups: (i) classes t-22506, t-15459, t-22508, t-22503 and (ii) t-22504, t-22507, t-15579. Class t-5504 appears in both subtrees.

When looking at the confusion matrix, the classes with the largest error are t-15459 and t-15579. They both appear only once in the leaf nodes. If we look at walk 14, the parent of the leaf node with class t-15459, we see that not finding the walk is more informative for class t-22508 than finding the walk for class t-15459. As a matter of fact, walk 14 can only be found in 3 of the 40 training entities of class t-15459. A higher depth will most likely be required in order to classify entities of that class accurately. Similarly, walk 9, the parent of the only leaf node with class t-15579, can only be found in 26 out of 93 training entities.

#### MUTAG

For the final data set, we demonstrate how insights can be still be retrieved from the less interpretable approaches, the Transform & Forest approach. We first apply the feature transform in order to extract 10000 different walks. Of these walks, 9463 of them only appear once in the neighborhood of an entity and are therefore removed. This results in a 537-dimensional binary feature vector for each of the train and test entities. Using these feature vectors, a LR classifier with Lasso regularization is fit. The accuracy of this classifier, on the test set, is 79.41%. Due to the Lasso regularization, 302 of the 537 coefficients have been set to 0. The interpretation of a coefficient *x*_*i*_ is that if the walk corresponding to that coefficient is found in the neighborhood of an entity, the prediction (before applying a sigmoid function to it) will increase with *x*_*i*_. We can therefore inspect the walks that have corresponding coefficients with the highest (positive) values or lowest (negative) values. The walks with the highest positive coefficients are:

- root -> * -> * -> * -> 0.016 (coef=44.19)
- root -> * -> * -> * -> * -> * -> 0.016 (coef=42.55)
- root -> * -> * -> * -> 0.588 (coef=18.41)
- root -> * -> * -> * -> -0.006 (coef=17.22)

If one of these walks is present in the neighborhood, the probability of being positive increased. On the other hand, we have the following walks associated with the lowest (negative) values:

- root -> * -> * -> * -> * -> * -> 0.146 (coef= −15.27)
- root -> * -> * -> * -> 0.016 (coef= −12.95)
- root -> * -> * -> * -> 0.027 (coef= −11.95)
- root -> * -> * -> * -> Alcohol (coef= −11.54)

These walks decrease the probability of being positive.

We repeat this analysis for the forest approach. We fit a forest of 25 different trees, each on 50% of the vertices in the graph with no maximum depth. We can now inspect the walks in the root nodes of the 25 trees, as these are the most important ones. The accuracy of the decision tree of this model is equal to 75%. Additionally, walks in the entire tree can be inspected, and a metric that takes into account the decrease in information gain or the position within the tree can be used to measure its importance. In the 25 root nodes, a total of 6 unique walks can be found, which are displayed below with their corresponding count:

- root -> * -> * -> * -> * -> * -> Carbon-10 (count=8)
- root -> * -> * -> * -> Carbon-10 (count=7)
- root -> * -> Five_ring (count=6)
- root -> * -> * -> * -> Carbon-16 (count=2)
- root -> * -> * -> * -> Ester (count=1)
- root -> * -> * -> * -> Non_ar_hetero_5_ring (count=1)

It appears that the presence of Carbon-10 (either 4 or 6 hops away from the entity) is very informative.

## Discussion

### Predictive performance

The results of Table 2, obtained in “Results” section, clearly show that all three of the proposed techniques are competitive to the current state-of-the-art for node classification in KGs. The Tree approach is only slightly worse than both techniques for *AIFB* and *AM*, while being better than RDF2VEC for *MUTAG* and outperforming both techniques for *BGS*. The Forest approach outperforms the two techniques on *BGS* and *MUTAG*. The Transform approach achieves the best predictive performance, achieving state-of-the-art results on three of the four data sets when the feature vectors are provided to both a LR and RF classifier. Only on the *AIFB* data set, the R-GCN outperforms all our proposed techniques. We speculate that this is due to the fact that the graph for *AIFB* is very dense, with a high average degree for the nodes within the immediate neighborhood of the training entities. The average degree of the entities that are 1 hop away from the training entities in the KG is equal to 11.61 for the *AIFB*, as opposed to 2.26, 3.68 and 10.57 for *MUTAG*, *BGS* and *AM* respectively. The R-GCN is able to efficiently aggregate information of these neighborhoods in an iterative fashion that implicitly allows the model to capture complex interactions that are not picked up by our techniques. The fact that the increase in predictive performance in *AM* is the smallest, can most likely be attributed to the same reason.

It should further be noted that accuracy is often not the ideal metric to measure the predictive performance with. Although it is one of the most intuitive metrics, it has several disadvantages such as skewness when data is imbalanced. Nevertheless, the accuracy metric is the only one allowing for comparison to related work, as that metric was used in those studies. Moreover, the used data sets are merely benchmark data sets and the goal is solely to compare the performance of our algorithms with the current state-of-the-art. We recommend using different performance metrics, which should be tailored to the specific use case. An example is using the area under the receiver operating characteristic curve (AUC) in combination with precision and recall at a certain probability threshold for medical data sets.

### Runtime

From the results provided in Table 3, we can see that the Transform technique is faster than the Tree approach, when the number of classes, which determines the number of passes over the entire data set, in the classification problem is low. For the Forest approach, we see the highest runtimes with a large variance caused by the fact that the tuned hyper-parameters were often different over the runs due to random partitioning of the cross-validation applied on the training data. Hyper-parameters such as the maximal depth of all the trees, and the number of trees in the forest have a large impact on the runtime.

### Comparison

For completeness, we provide an estimated ranking of the three proposed techniques across three dimensions: (i) computational runtime, (ii) interpretability, and (iii) predictive performance. The comparison is given in Table 4. The rankings are estimated based on the experience of the authors and based on the presented results. When interpretability and transparency matter, the decision tree technique is the most suitable candidate. Alternatively, when excellent predictive performance is of importance, the RF and feature transform techniques are preferable. Of these two, the feature transform approach is the fastest option.

**Table 4:** A comparison of the three proposed techniques across different dimensions

	Decision Tree	Random Forest	Feature Transform
Runtime	+	+	++
Interpretability	+++	+	++^∗^
Predictive performance	+	++	+++

^∗^ the feature transform approach is only interpretable if the model where the features are fed to is interpretable

## Conclusion and future work

In this paper, we presented an algorithm that allows mining for a specific type of walks that are informative for certain (groups of) classes, in the context of node classification for KGs. Moreover, we show that this algorithm is a good basis for a predictive model, when used in combination with one of three different techniques proposed in this work. Experiments on four KG benchmark data sets show that our proposed approaches outperform the current state-of-the-art while, in contrast to these techniques, being completely interpretable. This is of great importance for applications situated in critical domains.

It should be noted that we only focused on a very specific type of walk in this study, which allows for very efficient mining, but has a somewhat limited expressiveness and is less interpretable when compared to walks without wildcards. Nevertheless, by using multiple of these walks, good predictive performances can be achieved, as we demonstrated empirically. Future work should focus on algorithms that mine more expressive walks, e.g. by filling in some of the wildcards on the walk or by replacing nodes by subgraphs, while still being efficient. Moreover, the size of the data sets used in this study was rather moderate. An evaluation on larger data sets in terms of computational time and predictive performance would therefore be an interesting future step.

## Data Availability

An implementation of the proposed algorithms, and code to reproduce the experiments, are available on Github^1^. The benchmark data sets used for our experiments are available from the original authors^2^.

## Abbreviations

KG: Knowledge graph
LR: Logistic regression
RF: Random forest
R-GCN: Relational graph convolutional network
RDF: Resource description framework
